# Bis[1-(2,3-di­methyl­phen­yl)piperazine-1,4-diium] bis­(oxonium) cyclo­hexa­phosphate dihydrate

**DOI:** 10.1107/S1600536813016759

**Published:** 2013-06-22

**Authors:** Iness Ameur, Sonia Abid, Salem S Al-Deyab, Mohamed Rzaigui

**Affiliations:** a Laboratoire de Chimie des Matériaux, Faculté des Sciences de Bizerte, 7021 Zarzouna Bizerte, Tunisia; b Petrochemical Research Chair, College of Science, King Saud University, Riyadh, Saudi Arabia

## Abstract

In the title compound, 2C_12_H_20_N_2_^2+^·2H_3_O^+^·P_6_O_18_^6−^·2H_2_O, a protonated water mol­ecule bridges the centrosymmetrical anionic P_6_O_18_ ring *via* O—H⋯O hydrogen bonds. The centrosymmetric hydrogen-bonded rings formed by four oxonium cations and four phosphate anions can be described by an *R*_4_^8^(36) graph-set motif. The ring motifs are connected by hydrogen bonds into inorganic layers perpendicular to [100]. The 1-(2,3-di­methyl­phen­yl)piperazine-1,4-diium cations are located between the layers, compensating their negative charge and establishing N—H⋯O hydrogen bonds with the O atoms of the anionic framework.

## Related literature

For background to the chemistry of cyclo­hexa­phosphate, see: Durif (1995 ▶); Amri *et al.* (2008 ▶); Marouani *et al.* (2010 ▶). For applications of piperazine derivatives, see: Kaur *et al.* (2010 ▶); Eswaran *et al.* (2010 ▶); Chou *et al.* (2010 ▶); Chen *et al.* (2004 ▶); Shingalapur *et al.* (2009 ▶). For related structures with cyclo­hexa­phosphate rings, see: Abid *et al.* (2011 ▶); Ameur *et al.* (2013 ▶); Amri *et al.* (2009 ▶). For related structures with 1-phenyl­piperazine-1,4-diium salts, see: Marouani *et al.* (2010 ▶); Ben Gharbia *et al.* (2005 ▶). For puckering parameters, see: Cremer & Pople (1975 ▶). For graph-set descriptions of hydrogen-bond ring motifs, see: Bernstein *et al.* (1995 ▶). For the synthesis of the precursor, see: Schülke & Kayser (1985 ▶). 
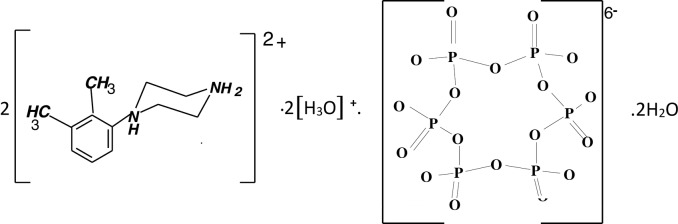


## Experimental

### 

#### Crystal data

2C_12_H_20_N_2_^2+^·2H_3_O^+^·P_6_O_18_^6−^·2H_2_O
*M* *_r_* = 932.50
Monoclinic, 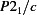

*a* = 8.630 (6) Å
*b* = 14.495 (4) Å
*c* = 17.072 (3) Å
β = 114.93 (4)°
*V* = 1936.6 (15) Å^3^
*Z* = 2
Ag *K*α radiation
λ = 0.56085 Å
μ = 0.20 mm^−1^
*T* = 293 K
0.60 × 0.40 × 0.10 mm

#### Data collection

Nonius MACH-3 diffractometer
Absorption correction: refined from Δ*F* (Walker & Stuart, 1983 ▶) *T*_min_ = 0.892, *T*_max_ = 0.981
12060 measured reflections
9442 independent reflections
5475 reflections with *I* > 2σ(*I*)
*R*_int_ = 0.031
2 standard reflections every 120 min intensity decay: none

#### Refinement

*R*[*F* ^2^ > 2σ(*F* ^2^)] = 0.055
*wR*(*F* ^2^) = 0.152
*S* = 0.99
9442 reflections
253 parameters
H-atom parameters constrained
Δρ_max_ = 0.87 e Å^−3^
Δρ_min_ = −0.67 e Å^−3^

### 

Data collection: *CAD-4 EXPRESS* (Enraf–Nonius, 1994 ▶); cell refinement: *CAD-4 EXPRESS*; data reduction: *XCAD4* (Harms & Wocadlo, 1996 ▶); program(s) used to solve structure: *SHELXS97* (Sheldrick, 2008 ▶); program(s) used to refine structure: *SHELXL97* (Sheldrick, 2008 ▶); molecular graphics: *ORTEP-3 for Windows* (Farrugia, 2012 ▶); software used to prepare material for publication: *WinGX* (Farrugia, 2012 ▶).

## Supplementary Material

Crystal structure: contains datablock(s) I, global. DOI: 10.1107/S1600536813016759/jj2168sup1.cif

Structure factors: contains datablock(s) I. DOI: 10.1107/S1600536813016759/jj2168Isup2.hkl

Additional supplementary materials:  crystallographic information; 3D view; checkCIF report

## Figures and Tables

**Table 1:** Hydrogen-bond geometry (Å, °)

*D*—H⋯*A*	*D*—H	H⋯*A*	*D*⋯*A*	*D*—H⋯*A*
O*W*1—H1*W*1⋯O9	0.79	1.84	2.614 (3)	166
O*W*1—H2*W*1⋯O2^i^	0.86	1.97	2.781 (3)	159
O*W*2—H1*W*2⋯O8	0.82	1.69	2.487 (2)	167
O*W*2—H2*W*2⋯O*W*1^ii^	0.80	1.77	2.503 (3)	152
O*W*2—H3*W*2⋯O6^iii^	0.85	1.64	2.481 (2)	178
N1—H1⋯O1	0.91	1.82	2.690 (2)	160
N2—H2*A*⋯O5^iv^	0.90	1.87	2.714 (2)	156
N2—H2*B*⋯O2^v^	0.90	2.10	2.858 (3)	142
N2—H2*B*⋯O5^v^	0.90	2.28	2.916 (3)	127

Symmetry codes: (i) 
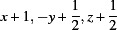
; (ii) 
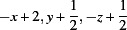
; (iii) 
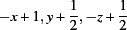
; (iv) 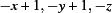; (v) 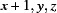.

**Fig. 1.**
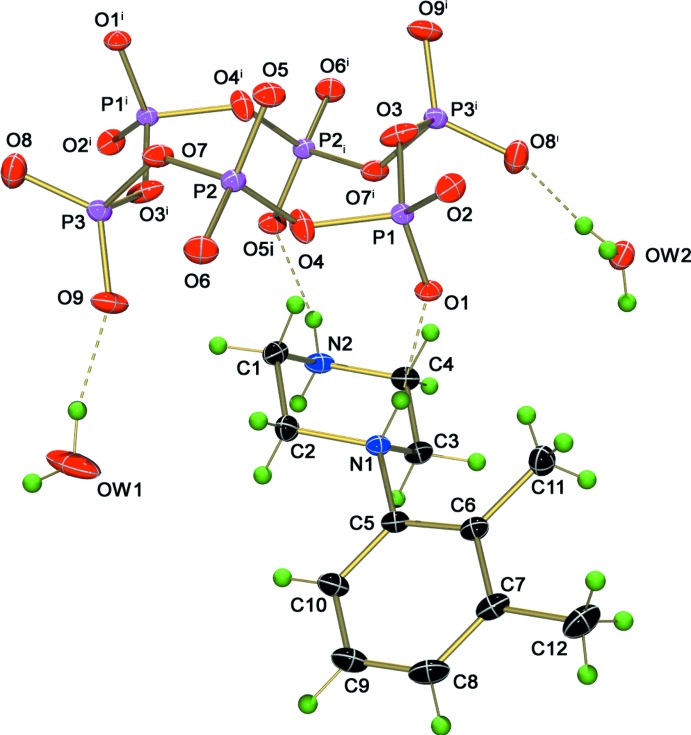
The molecular structure of (I) with 50% probability displacement ellipsoids. Dashed lines indicate O—H···O and N—H···O hydrogen bonds.

**Fig. 2.**
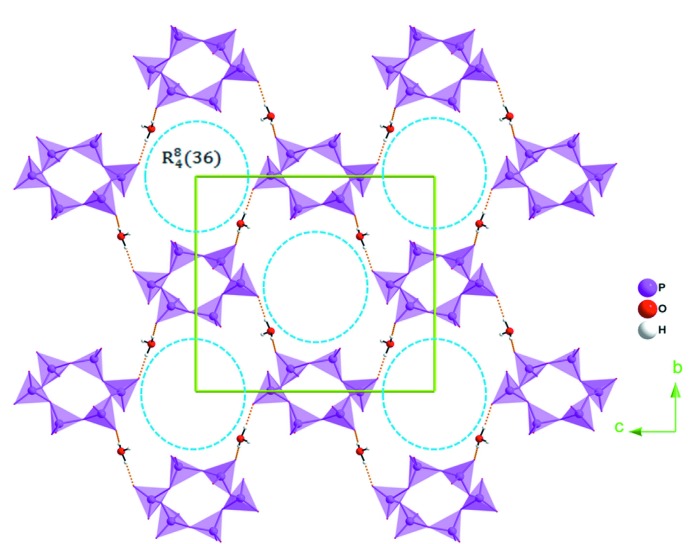
Fig, 2. Projection along the a axis, of an inorganic layer in the structure of (I). The dashed circles highlight the R48(36) centrosymmetric motifs.

**Fig. 3.**
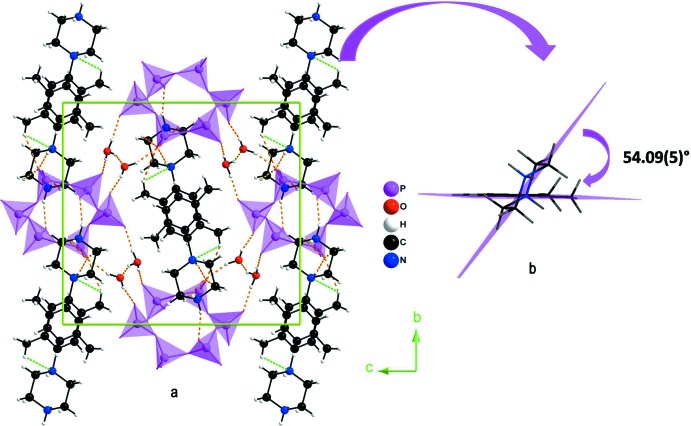
(a) The three-dimensional network of (I), projected along the aaxis. (b) Relative orientation of the rings aryl and piperazine in the 1-(2,3-Dimethylphenyl)piperazine-1,4-diium cation.

## supplementary crystallographic information

### Comment

The literature reports several cyclohexaphosphates of organic cations and/or inorganic cations (Durif,1995). However, cyclohexaphosphates of mixed cations associating the oxoniumion are still relatively very limited. Up to now, only two examples have been known and structurally characterized (Amri *et al.*, 2008, Marouani *et al.*,2010). In this work, we report the preparation and the structural investigation of a new organic oxonium cyclohexaphospohate, 2(C_12_H_20_N_2_)^2+^.2H_3_O^+^.P_6_O_18_^6-^. 2H_2_O, (I), where the organic species is the piperazinium group. Piperazine derivatives have wide range of applications in pharmaceuticals as antimalarial (Kaur *et al.*, 2010), anti-tuberculosis (Eswaran *et al.*, 2010), antitumor (Chou *et al.*, 2010), anticancer (Chen *et al.*, 2004) and antiviral (Shingalapur *et al.*, 2009) agents.

The asymmetric unit of (I) includes one-half of the P_6_O_18_ ring lying on an inversion center (1/2, 1/2, 0), one 1-(2,3-dimethylphenyl) piperazine-1,4-diium cation, one hydronium cation and one water molecule (Fig.1). As shown in Fig.2, the hydronium cations (OW2) bridge the anionic ring to form 2-D corrugated layers, located at *x* = 1/2 and parallel to the *bc*-plane. The result of these interactions is the formation of a 36-membered ring with an *R*_4_^8^(36) graph-set motif (Bernstein *et al.*, 1995). The centre of 36-membered ring is situated on a crystallographic centre of symmetry. Inside these layers, the phosphoric rings display a chair conformation with geometrical characteristics that show no significant difference in deviation from those observed in other cyclohexaphosphates having the same internal symmetry -1 (Amri *et al.*, 2009; Abid *et al.*, 2011; Ameur *et al.*, 2013). The anchorage of the water molecule OW1 and the organic cations is made by short and long H-bonds, ensuring the interconnection between layers, and thus giving rise to a three-dimensional network. The benzyl ring (C5—C10) is essentially planar with an r.m.s. deviation of 0.0047 Å and is orientated at an angle of 54.09 (5)° with respect to the piperazine ring (Fig.3). The piperazine (N1–N2/C1–C4) ring adopts a chair conformation [puckering parameters: Q_T_ = 0.577 (2) Å, θ = 0.7 (2)° and φ = 326 (12) (Cremer & Pople, 1975)] with atoms N1 and N2 deviating by -0.683 (2) and 0.657 (2) Å from the least-squares plane defined by the remaining atoms in the ring. The interatomic bond lengths (C—C,*N*—C) and angles in (C—C—C,*C*—N—C) do not show significant deviation from those reported in a related 1-phenylpiperazine-1,4-diium salt (Ben Gharbia *et al.*, 2005). An extensive network of N—H···O and O—H···O hydrogen-bonding interactions link the components of the structure into a three-dimensional network (Fig. 3).

### Experimental

Crystals of the title compound were prepared by adding dropwise an ethanolic solution (5 ml) of 1-(2,3)dimethlphenylpiperazine (4 mmol) to an aqueous solution (10 ml) of cyclohexaphosphoric acid (2 mmol). The reaction mixture was stirred at room temperature for few minutes. X-ray quality crystals of the title compound appeared after a few days. The cyclohexaphosphoric acid H_6_P_6_O_18_, was produced from Li_6_P_6_O_18_.6H_2_O, prepared according to the procedure of Schülke and Kayser (Schülke & Kayser, 1985), through an ion-exchange resin in H-state (Amberlite IR 120).

### Refinement

H1W1, H1W2, H2W1, H2W2 and H3W2 were located by Fourier maps and refined as riding in their as-found relative positions with *U*_iso_(H) = 1.5*U*_eq_(O). All remaining H atoms were placed in their calculated positions and then refined using the riding model with atom-H lengths of 0.93 Å (CH), 0.97 Å (CH_2_), 0.96 Å (CH_3_), 0.91 Å (NH) and 0.90 Å (NH_3_). *U*_iso_ were set to 1.2 (CH, CH_2_), 1.5 (CH_3_) or 1.20 (NH) times *U*_eq_ of the parent atom.

### Crystal data

**Table:**

2C_12_H_20_N_2_^2+^·2H_3_O^+^·P_6_O_18_^6^^−^·2H_2_O	*F*(000) = 976
*M**_r_* = 932.50	*D*_x_ = 1.599 Mg m^−^^3^
Monoclinic, *P*2_1_/*c*	Ag *K*α radiation, λ = 0.56085 Å
Hall symbol: -P 2ybc	Cell parameters from 25 reflections
*a* = 8.630 (6) Å	θ = 9.3–10.5°
*b* = 14.495 (4) Å	µ = 0.20 mm^−^^1^
*c* = 17.072 (3) Å	*T* = 293 K
β = 114.93 (4)°	Prism, colourless
*V* = 1936.6 (15) Å^3^	0.60 × 0.40 × 0.10 mm
*Z* = 2	

### Data collection

**Table:**

Nonius MACH-3 diffractometer	5475 reflections with *I* > 2σ(*I*)
Radiation source: fine-focus sealed tube	*R*_int_ = 0.031
Graphite monochromator	θ_max_ = 28.0°, θ_min_ = 2.1°
non–profiled ω scans	*h* = −14→14
Absorption correction: part of the refinement model (Δ*F*) (Walker & Stuart, 1983)	*k* = −24→2
*T*_min_ = 0.892, *T*_max_ = 0.981	*l* = −28→16
12060 measured reflections	2 standard reflections every 120 min
9442 independent reflections	intensity decay: none

### Refinement

**Table:**

Refinement on *F*^2^	Primary atom site location: structure-invariant direct methods
Least-squares matrix: full	Secondary atom site location: difference Fourier map
*R*[*F*^2^ > 2σ(*F*^2^)] = 0.055	Hydrogen site location: inferred from neighbouring sites
*wR*(*F*^2^) = 0.152	H-atom parameters constrained
*S* = 0.99	*w* = 1/[σ^2^(*F*_o_^2^) + (0.0763*P*)^2^] where *P* = (*F*_o_^2^ + 2*F*_c_^2^)/3
9442 reflections	(Δ/σ)_max_ < 0.001
253 parameters	Δρ_max_ = 0.87 e Å^−^^3^
0 restraints	Δρ_min_ = −0.67 e Å^−^^3^
0 constraints	

### Special details

**Table:**

Geometry. All e.s.d.'s (except the e.s.d. in the dihedral angle between two l.s. planes) are estimated using the full covariance matrix. The cell e.s.d.'s are taken into account individually in the estimation of e.s.d.'s in distances, angles and torsion angles; correlations between e.s.d.'s in cell parameters are only used when they are defined by crystal symmetry. An approximate (isotropic) treatment of cell e.s.d.'s is used for estimating e.s.d.'s involving l.s. planes.
Refinement. Refinement of *F*^2^ against ALL reflections. The weighted *R*-factor *wR* and goodness of fit *S* are based on *F*^2^, conventional *R*-factors *R* are based on *F*, with *F* set to zero for negative *F*^2^. The threshold expression of *F*^2^ > σ(*F*^2^) is used only for calculating *R*-factors(gt) *etc*. and is not relevant to the choice of reflections for refinement. *R*-factors based on *F*^2^ are statistically about twice as large as those based on *F*, and *R*- factors based on ALL data will be even larger.

### Fractional atomic coordinates and isotropic or equivalent isotropic displacement parameters (Å^2^)

**Table:**

	*x*	*y*	*z*	*U*_iso_*/*U*_eq_	
P1	0.31249 (5)	0.34945 (3)	−0.07303 (3)	0.02004 (9)	
P2	0.37416 (6)	0.39535 (3)	0.10432 (3)	0.02228 (10)	
P3	0.69777 (6)	0.49655 (3)	0.18679 (3)	0.02396 (10)	
O1	0.42651 (16)	0.30040 (10)	−0.10364 (9)	0.0287 (3)	
O2	0.14736 (17)	0.30794 (10)	−0.08650 (11)	0.0345 (3)	
O3	0.2778 (2)	0.45146 (10)	−0.11034 (11)	0.0385 (4)	
O4	0.42266 (17)	0.36921 (13)	0.02683 (9)	0.0403 (4)	
O5	0.19648 (16)	0.42960 (10)	0.07175 (10)	0.0321 (3)	
O6	0.4264 (2)	0.31760 (10)	0.16614 (10)	0.0351 (3)	
O7	0.49427 (17)	0.48166 (9)	0.14480 (10)	0.0325 (3)	
O8	0.7354 (2)	0.55904 (12)	0.26036 (10)	0.0448 (4)	
O9	0.7852 (2)	0.40717 (10)	0.19877 (12)	0.0419 (4)	
OW1	1.0380 (3)	0.2895 (2)	0.25912 (15)	0.0936 (10)	
H1W1	0.9712	0.3308	0.2474	0.140*	
H2W1	1.0790	0.2734	0.3123	0.140*	
OW2	0.79099 (19)	0.72073 (11)	0.31371 (10)	0.0372 (3)	
H1W2	0.7581	0.6706	0.2912	0.056*	
H2W2	0.8182	0.7360	0.2764	0.056*	
H3W2	0.7184	0.7547	0.3209	0.056*	
C1	0.8112 (2)	0.37054 (13)	0.00581 (13)	0.0295 (4)	
H1A	0.6935	0.3897	−0.0121	0.035*	
H1B	0.8824	0.4074	0.0554	0.035*	
C2	0.8290 (3)	0.26990 (14)	0.03063 (13)	0.0302 (4)	
H2C	0.9484	0.2523	0.0532	0.036*	
H2D	0.7907	0.2605	0.0759	0.036*	
C3	0.7820 (3)	0.22745 (13)	−0.11665 (13)	0.0282 (4)	
H3A	0.7134	0.1904	−0.1667	0.034*	
H3B	0.9005	0.2093	−0.0976	0.034*	
C4	0.7625 (3)	0.32817 (13)	−0.14143 (14)	0.0299 (4)	
H4A	0.8012	0.3384	−0.1865	0.036*	
H4B	0.6429	0.3454	−0.1640	0.036*	
C5	0.7366 (2)	0.10980 (12)	−0.02396 (12)	0.0258 (3)	
C6	0.6060 (2)	0.05226 (13)	−0.07716 (12)	0.0265 (3)	
C7	0.6213 (3)	−0.04254 (14)	−0.05737 (14)	0.0335 (4)	
C8	0.7617 (3)	−0.07472 (15)	0.01414 (17)	0.0421 (5)	
H8	0.7708	−0.1374	0.0270	0.051*	
C9	0.8877 (3)	−0.01554 (16)	0.06639 (17)	0.0433 (5)	
H9	0.9801	−0.0383	0.1144	0.052*	
C10	0.8771 (3)	0.07749 (16)	0.04755 (15)	0.0364 (5)	
H10	0.9623	0.1178	0.0821	0.044*	
C11	0.4528 (3)	0.08562 (15)	−0.15409 (13)	0.0354 (4)	
H13	0.4447	0.1515	−0.1513	0.053*	
H11	0.3517	0.0578	−0.1544	0.053*	
H14	0.4639	0.0690	−0.2059	0.053*	
C12	0.4877 (4)	−0.10878 (16)	−0.11266 (19)	0.0477 (6)	
H122	0.3770	−0.0866	−0.1210	0.072*	
H121	0.5077	−0.1680	−0.0850	0.072*	
H123	0.4924	−0.1144	−0.1676	0.072*	
N1	0.72640 (18)	0.20991 (10)	−0.04534 (10)	0.0227 (3)	
H1	0.6150	0.2273	−0.0649	0.027*	
N2	0.8629 (2)	0.38610 (11)	−0.06582 (12)	0.0298 (3)	
H2A	0.8476	0.4459	−0.0813	0.036*	
H2B	0.9746	0.3729	−0.0475	0.036*	

### Atomic displacement parameters (Å^2^)

**Table:**

	*U*^11^	*U*^22^	*U*^33^	*U*^12^	*U*^13^	*U*^23^
P1	0.01473 (16)	0.02149 (19)	0.0252 (2)	−0.00054 (15)	0.00971 (15)	−0.00045 (17)
P2	0.02042 (19)	0.02102 (19)	0.0271 (2)	−0.00034 (15)	0.01164 (17)	−0.00057 (17)
P3	0.02164 (19)	0.02002 (19)	0.0283 (2)	0.00048 (16)	0.00867 (17)	0.00217 (17)
O1	0.0217 (6)	0.0296 (7)	0.0363 (7)	0.0055 (5)	0.0137 (5)	−0.0021 (6)
O2	0.0221 (6)	0.0335 (7)	0.0522 (9)	−0.0093 (5)	0.0197 (6)	−0.0100 (7)
O3	0.0558 (10)	0.0228 (6)	0.0560 (10)	0.0102 (6)	0.0420 (8)	0.0084 (7)
O4	0.0196 (6)	0.0758 (12)	0.0257 (7)	0.0018 (7)	0.0099 (5)	−0.0072 (8)
O5	0.0205 (6)	0.0297 (7)	0.0475 (8)	0.0007 (5)	0.0157 (6)	−0.0001 (6)
O6	0.0388 (8)	0.0270 (7)	0.0420 (8)	0.0021 (6)	0.0195 (7)	0.0086 (6)
O7	0.0224 (6)	0.0209 (6)	0.0518 (9)	−0.0013 (5)	0.0132 (6)	−0.0039 (6)
O8	0.0574 (10)	0.0368 (9)	0.0331 (8)	−0.0078 (8)	0.0122 (7)	−0.0075 (7)
O9	0.0328 (8)	0.0290 (7)	0.0603 (10)	0.0119 (6)	0.0160 (7)	0.0105 (7)
OW1	0.0987 (18)	0.134 (2)	0.0705 (14)	0.0923 (17)	0.0579 (14)	0.0573 (15)
OW2	0.0349 (7)	0.0385 (8)	0.0389 (8)	−0.0028 (6)	0.0162 (7)	−0.0106 (7)
C1	0.0236 (8)	0.0264 (8)	0.0393 (10)	−0.0026 (7)	0.0139 (8)	−0.0055 (8)
C2	0.0283 (9)	0.0289 (9)	0.0304 (9)	−0.0021 (7)	0.0093 (7)	−0.0018 (8)
C3	0.0316 (9)	0.0249 (8)	0.0363 (10)	0.0015 (7)	0.0223 (8)	0.0021 (7)
C4	0.0297 (9)	0.0280 (9)	0.0369 (10)	0.0011 (7)	0.0188 (8)	0.0048 (8)
C5	0.0272 (8)	0.0216 (8)	0.0324 (9)	0.0038 (6)	0.0164 (7)	0.0035 (7)
C6	0.0323 (9)	0.0245 (8)	0.0299 (9)	0.0023 (7)	0.0200 (7)	0.0014 (7)
C7	0.0459 (11)	0.0222 (8)	0.0442 (11)	0.0000 (8)	0.0305 (10)	−0.0008 (8)
C8	0.0526 (14)	0.0252 (9)	0.0598 (15)	0.0097 (9)	0.0346 (12)	0.0128 (10)
C9	0.0426 (12)	0.0339 (11)	0.0497 (13)	0.0126 (9)	0.0158 (10)	0.0146 (10)
C10	0.0310 (10)	0.0325 (10)	0.0413 (11)	0.0075 (8)	0.0110 (9)	0.0076 (9)
C11	0.0364 (10)	0.0329 (10)	0.0335 (10)	−0.0052 (8)	0.0115 (8)	−0.0028 (8)
C12	0.0568 (15)	0.0290 (10)	0.0662 (17)	−0.0098 (10)	0.0346 (13)	−0.0116 (11)
N1	0.0199 (6)	0.0212 (6)	0.0289 (7)	0.0015 (5)	0.0120 (6)	0.0014 (6)
N2	0.0210 (6)	0.0226 (7)	0.0483 (10)	0.0014 (6)	0.0170 (7)	0.0031 (7)

### Geometric parameters (Å, º)

**Table:**

P1—O2	1.4729 (16)	C3—H3A	0.9700
P1—O1	1.4766 (15)	C3—H3B	0.9700
P1—O3	1.5878 (15)	C4—N2	1.476 (3)
P1—O4	1.5894 (16)	C4—H4A	0.9700
P2—O6	1.4785 (15)	C4—H4B	0.9700
P2—O5	1.4792 (17)	C5—C6	1.388 (3)
P2—O7	1.5855 (15)	C5—C10	1.390 (3)
P2—O4	1.5924 (15)	C5—N1	1.490 (2)
P3—O8	1.4696 (17)	C6—C7	1.408 (3)
P3—O9	1.4696 (15)	C6—C11	1.497 (3)
P3—O3^i^	1.5964 (15)	C7—C8	1.389 (3)
P3—O7	1.6073 (19)	C7—C12	1.491 (3)
O3—P3^i^	1.5964 (15)	C8—C9	1.377 (4)
OW1—H1W1	0.7950	C8—H8	0.9300
OW1—H2W1	0.8565	C9—C10	1.380 (3)
OW2—H1W2	0.8154	C9—H9	0.9300
OW2—H2W2	0.7977	C10—H10	0.9300
OW2—H3W2	0.8458	C11—H13	0.9600
C1—N2	1.485 (3)	C11—H11	0.9600
C1—C2	1.509 (3)	C11—H14	0.9600
C1—H1A	0.9700	C12—H122	0.9600
C1—H1B	0.9700	C12—H121	0.9600
C2—N1	1.501 (2)	C12—H123	0.9600
C2—H2C	0.9700	N1—H1	0.9100
C2—H2D	0.9700	N2—H2A	0.9000
C3—N1	1.506 (2)	N2—H2B	0.9000
C3—C4	1.509 (3)		
			
O2—P1—O1	119.56 (9)	N2—C4—H4B	109.6
O2—P1—O3	108.13 (9)	C3—C4—H4B	109.6
O1—P1—O3	110.35 (8)	H4A—C4—H4B	108.1
O2—P1—O4	110.06 (9)	C6—C5—C10	122.63 (18)
O1—P1—O4	106.25 (9)	C6—C5—N1	118.49 (16)
O3—P1—O4	100.89 (10)	C10—C5—N1	118.88 (17)
O6—P2—O5	118.69 (9)	C5—C6—C7	117.46 (18)
O6—P2—O7	110.10 (9)	C5—C6—C11	123.56 (17)
O5—P2—O7	106.42 (9)	C7—C6—C11	118.97 (18)
O6—P2—O4	107.69 (10)	C8—C7—C6	119.8 (2)
O5—P2—O4	111.19 (9)	C8—C7—C12	119.7 (2)
O7—P2—O4	101.38 (9)	C6—C7—C12	120.5 (2)
O8—P3—O9	120.58 (11)	C9—C8—C7	121.2 (2)
O8—P3—O3^i^	110.49 (10)	C9—C8—H8	119.4
O9—P3—O3^i^	107.03 (9)	C7—C8—H8	119.4
O8—P3—O7	105.68 (10)	C8—C9—C10	120.0 (2)
O9—P3—O7	110.14 (9)	C8—C9—H9	120.0
O3^i^—P3—O7	101.25 (9)	C10—C9—H9	120.0
P1—O3—P3^i^	134.42 (10)	C9—C10—C5	118.8 (2)
P1—O4—P2	133.32 (10)	C9—C10—H10	120.6
P2—O7—P3	133.71 (9)	C5—C10—H10	120.6
H1W1—OW1—H2W1	113.9	C6—C11—H13	109.5
H1W2—OW2—H2W2	91.8	C6—C11—H11	109.5
H1W2—OW2—H3W2	117.1	H13—C11—H11	109.5
H2W2—OW2—H3W2	116.2	C6—C11—H14	109.5
N2—C1—C2	110.34 (16)	H13—C11—H14	109.5
N2—C1—H1A	109.6	H11—C11—H14	109.5
C2—C1—H1A	109.6	C7—C12—H122	109.5
N2—C1—H1B	109.6	C7—C12—H121	109.5
C2—C1—H1B	109.6	H122—C12—H121	109.5
H1A—C1—H1B	108.1	C7—C12—H123	109.5
N1—C2—C1	111.36 (16)	H122—C12—H123	109.5
N1—C2—H2C	109.4	H121—C12—H123	109.5
C1—C2—H2C	109.4	C5—N1—C2	113.59 (15)
N1—C2—H2D	109.4	C5—N1—C3	110.88 (14)
C1—C2—H2D	109.4	C2—N1—C3	109.11 (14)
H2C—C2—H2D	108.0	C5—N1—H1	107.7
N1—C3—C4	110.72 (15)	C2—N1—H1	107.7
N1—C3—H3A	109.5	C3—N1—H1	107.7
C4—C3—H3A	109.5	C4—N2—C1	111.35 (15)
N1—C3—H3B	109.5	C4—N2—H2A	109.4
C4—C3—H3B	109.5	C1—N2—H2A	109.4
H3A—C3—H3B	108.1	C4—N2—H2B	109.4
N2—C4—C3	110.49 (16)	C1—N2—H2B	109.4
N2—C4—H4A	109.6	H2A—N2—H2B	108.0
C3—C4—H4A	109.6		
			
O2—P1—O3—P3^i^	−116.25 (16)	C5—C6—C7—C8	1.2 (3)
O1—P1—O3—P3^i^	16.22 (19)	C11—C6—C7—C8	−179.47 (19)
O4—P1—O3—P3^i^	128.25 (16)	C5—C6—C7—C12	−178.77 (18)
O2—P1—O4—P2	−31.2 (2)	C11—C6—C7—C12	0.6 (3)
O1—P1—O4—P2	−162.03 (16)	C6—C7—C8—C9	−0.2 (3)
O3—P1—O4—P2	82.82 (18)	C12—C7—C8—C9	179.7 (2)
O6—P2—O4—P1	114.23 (17)	C7—C8—C9—C10	−0.8 (4)
O5—P2—O4—P1	−17.4 (2)	C8—C9—C10—C5	0.8 (4)
O7—P2—O4—P1	−130.16 (17)	C6—C5—C10—C9	0.2 (3)
O6—P2—O7—P3	53.82 (17)	N1—C5—C10—C9	−178.9 (2)
O5—P2—O7—P3	−176.32 (13)	C6—C5—N1—C2	157.24 (16)
O4—P2—O7—P3	−59.98 (16)	C10—C5—N1—C2	−23.6 (2)
O8—P3—O7—P2	−140.97 (14)	C6—C5—N1—C3	−79.5 (2)
O9—P3—O7—P2	−9.23 (18)	C10—C5—N1—C3	99.7 (2)
O3^i^—P3—O7—P2	103.78 (15)	C1—C2—N1—C5	−178.79 (15)
N2—C1—C2—N1	−56.9 (2)	C1—C2—N1—C3	57.0 (2)
N1—C3—C4—N2	58.0 (2)	C4—C3—N1—C5	176.94 (15)
C10—C5—C6—C7	−1.2 (3)	C4—C3—N1—C2	−57.2 (2)
N1—C5—C6—C7	177.91 (16)	C3—C4—N2—C1	−57.5 (2)
C10—C5—C6—C11	179.48 (19)	C2—C1—N2—C4	56.8 (2)
N1—C5—C6—C11	−1.4 (3)		

Symmetry code: (i) −*x*+1, −*y*+1, −*z*.

### Hydrogen-bond geometry (Å, º)

**Table:**

*D*—H···*A*	*D*—H	H···*A*	*D*···*A*	*D*—H···*A*
O*W*1—H1*W*1···O9	0.79	1.84	2.614 (3)	166
O*W*1—H2*W*1···O2^ii^	0.86	1.97	2.781 (3)	159
O*W*2—H1*W*2···O8	0.82	1.69	2.487 (2)	167
O*W*2—H2*W*2···O*W*1^iii^	0.80	1.77	2.503 (3)	152
O*W*2—H3*W*2···O6^iv^	0.85	1.64	2.481 (2)	178
N1—H1···O1	0.91	1.82	2.690 (2)	160
N2—H2*A*···O5^i^	0.90	1.87	2.714 (2)	156
N2—H2*B*···O2^v^	0.90	2.10	2.858 (3)	142
N2—H2*B*···O5^v^	0.90	2.28	2.916 (3)	127

Symmetry codes: (i) −*x*+1, −*y*+1, −*z*; (ii) *x*+1, −*y*+1/2, *z*+1/2; (iii) −*x*+2, *y*+1/2, −*z*+1/2; (iv) −*x*+1, *y*+1/2, −*z*+1/2; (v) *x*+1, *y*, *z*.

## References

[bb1] Abid, S., Al-Deyab, S. S. & Rzaigui, M. (2011). *Acta Cryst.* E**67**, m1549–m1550. 10.1107/S160053681104133X PMC3246971 22219791

[bb2] Ameur, I., Abid, S., Al-Deyab, S. S. & Rzaigui, M. (2013). *Acta Cryst.* E**69**, m305–m306. 10.1107/S1600536813011756 PMC3684873 23794975

[bb3] Amri, O., Abid, S. & Rzaigui, M. (2008). *Phosphorus Sulfur Silicon Relat. Elem.* **183**, 1996–2005.

[bb4] Amri, O., Abid, S. & Rzaigui, M. (2009). *Acta Cryst.* E**65**, o654. 10.1107/S1600536809006655 PMC2968505 21582302

[bb5] Ben Gharbia, I., Kefi, R., Rayes, A. & Ben Nasr, C. (2005). *Z. Kristallogr.* **220**, 333–334.

[bb6] Bernstein, J., David, R. E., Shimoni, L. & Chang, N. L. (1995). *Angew. Chem. Int. Ed. Engl.* **34**, 1555–1573.

[bb7] Chen, Y. L., Hung, H. M., Lu, C. M., Li, K. C. & Tzeng, C. C. (2004). *Bioorg. Med. Chem.* **12**, 6539–6546. 10.1016/j.bmc.2004.09.025 15556770

[bb8] Chou, L. C., Tsai, M. T., Hsu, M. H., Wang, S. H., Way, T. D., Huang, C. H., Lin, H. Y., Qian, K., Dong, Y., Lee, K. H., Huang, L. J. & Kuo, S. C. (2010). *J. Med. Chem.* **53**, 8047–8058. 10.1021/jm100780c 20973552

[bb9] Cremer, D. & Pople, J. A. (1975). *J. Am. Chem. Soc.* **97**, 1354–1358.

[bb10] Durif, A. (1995). In *Crystal Chemistry of Condensed Phosphates* New York and London: Plenum Press.

[bb11] Enraf–Nonius (1994). *CAD-4 EXPRESS* Enraf–Nonius, Delft, The Netherlands.

[bb12] Eswaran, S., Adhikari, A. V., Chowdhury, I. H., Pal, N. K. & Thomas, K. D. (2010). *Eur. J. Med. Chem.* **45**, 3374–3383. 10.1016/j.ejmech.2010.04.022 20537437

[bb13] Farrugia, L. J. (2012). *J. Appl. Cryst.* **45**, 849–854.

[bb14] Harms, K. & Wocadlo, S. (1996). *XCAD4* University of Marburg, Germany.

[bb15] Kaur, K., Jain, M., Reddy, R. P. & Jain, R. (2010). *Eur. J. Med. Chem.* **45**, 3245–3264. 10.1016/j.ejmech.2010.04.011 20466465

[bb16] Marouani, H., Rzaigui, M. & Al-Deyab, S. S. (2010). *Acta Cryst.* E**66**, o2613. 10.1107/S1600536810037001 PMC2983403 21587588

[bb17] Schülke, U. & Kayser, R. (1985). *Z. Anorg. Allg. Chem.* **531**, 167–175.

[bb18] Sheldrick, G. M. (2008). *Acta Cryst.* A**64**, 112–122. 10.1107/S0108767307043930 18156677

[bb19] Shingalapur, R. V., Hosamani, K. M. & Keri, R. S. (2009). *Eur. J. Med. Chem.* **44**, 4244–4248. 10.1016/j.ejmech.2009.05.021 19540630

[bb20] Walker, N. & Stuart, D. (1983). *Acta Cryst.* A**39**, 158–166.

